# SeqURE – a new copy-capture based method for sequencing of unknown Retroposition events

**DOI:** 10.1186/s13100-020-00228-6

**Published:** 2020-12-14

**Authors:** Alexander Y. Komkov, Shamil Z. Urazbakhtin, Maria V. Saliutina, Ekaterina A. Komech, Yuri A. Shelygin, Gaiaz A. Nugmanov, Vitaliy P. Shubin, Anastasia O. Smirnova, Mikhail Y. Bobrov, Alexey S. Tsukanov, Anastasia V. Snezhkina, Anna V. Kudryavtseva, Yuri B. Lebedev, Ilgar Z. Mamedov

**Affiliations:** 1 Shemyakin-Ovchinnikov Institute of Bioorganic Chemistry, Moscow, Russia; 2 Dmitry Rogachev National Medical and Research Center of Pediatric Hematology, Oncology and Immunology, Moscow, Russia; 3 Ryzhikh National Medical Research Centre for Coloproctology of the Ministry of Health of Russia, Moscow, Russia; 4 V.I. Kulakov National Medical Research Center for Obstetrics, Gynecology and Perinatology, Moscow, Russia; 5 Engelhardt Institute of Molecular Biology, Russian Academy of Sciences, Moscow, Russia; 6 Central European Institute of Technology, Masaryk University, Brno, Czech Republic

**Keywords:** Retroelements, Human genome, Insertional polymorphism, Copy capture, High-throughput sequencing

## Abstract

**Background:**

Retroelements (REs) occupy a significant part of all eukaryotic genomes including humans. The majority of retroelements in the human genome are inactive and unable to retrotranspose. Dozens of active copies are repressed in most normal tissues by various cellular mechanisms. These copies can become active in normal germline and brain tissues or in cancer, leading to new retroposition events. The consequences of such events and their role in normal cell functioning and carcinogenesis are not yet fully understood. If new insertions occur in a small portion of cells they can be found only with the use of specific methods based on RE enrichment and high-throughput sequencing. The downside of the high sensitivity of such methods is the presence of various artifacts imitating real insertions, which in many cases cannot be validated due to lack of the initial template DNA. For this reason, adequate assessment of rare (< 1%) subclonal cancer specific RE insertions is complicated.

**Results:**

Here we describe a new copy-capture technique which we implemented in a method called SeqURE for Sequencing Unknown of Retroposition Events that allows for efficient and reliable identification of new genomic RE insertions. The method is based on the capture of copies of target molecules (copy-capture), selective amplification and sequencing of genomic regions adjacent to active RE insertions from both sides. Importantly, the template genomic DNA remains intact and can be used for validation experiments. In addition, we applied a novel system for testing method sensitivity and precisely showed the ability of the developed method to reliably detect insertions present in 1 out of 100 cells and a substantial portion of insertions present in 1 out of 1000 cells. Using advantages of the method we showed the absence of somatic Alu insertions in colorectal cancer samples bearing tumor-specific L1HS insertions.

**Conclusions:**

This study presents the first description and implementation of the copy-capture technique and provides the first methodological basis for the quantitative assessment of RE insertions present in a small portion of cells.

**Supplementary Information:**

The online version contains supplementary material available at 10.1186/s13100-020-00228-6.

## Background

Retroelements (REs) in the human genome comprise more than 1.5 million copies that arise as a result of a process called retrotransposition. Three major classes of REs are LTR retroposons, LINEs, and SINEs that include the primate-specific Alu family. Only a tiny portion of REs is still capable of retrotransposition including more than one hundred of autonomous LINE-1 (L1) copies [1] and non-autonomous Alu and SVA that recruit the L1 enzymes for retrotransposition. The activity of these copies results in new L1, SVA and Alu insertions found in the genome of newborns [2, 3] and also leads to somatic insertions in cancer [4–7] or normal tissues [8–11]. These insertions can be neutral, can cause genetic disorders, or could promote the development of cancer. The role of RE insertions in the generation of neuronal plasticity during adult neurogenesis is also considered [12–14]. In the past decade, several methods for screening for new RE insertions have been developed. Most modern methods are based on high-throughput sequencing and can be divided into targeted and non-targeted. Non-targeted methods for new RE insertions discovery includes analysis of whole genome (WGS) or exome sequencing data with various software tools [2, 5, 15, 16]. This approach is quite reliable if a new insertion is supported by many different sequencing reads which means that WGS data have a high sequencing coverage. Insertions present in a low percentage of cells in a sample are detected with less confidence and must be supported by independent methods such as locus-specific PCR combined with Sanger sequencing. In most cases with standard WGS coverage non targeted methods allow to find insertions present in 25% of cells or more [16]. Targeted methods of RE insertion detection include enrichment in RE sequences or their unique genomic flanking regions (flanks) prior to sequencing [14, 17–22]. The enrichment methods are based on either hybridization to RE specific oligos or selective amplification of RE flanks. These methods are more sensitive in respect of the catching somatic insertion events and obviously are more cost-effective compared to WGS. However, all the existing enrichment techniques can generate various artificial sequences (chimeras) that mimic true RE insertions and lead to many false-positive results [23, 24]. Thus, independent confirmation of insertions by locus-specific PCR starting from initial genomic DNA samples with subsequent Sanger sequencing and identification of Target Site Duplication (TSD – the sign of true retrotransposition event) became a gold standard in somatic RE studies [23]. In the case of insertions present in the genome of very few cells in the sample, this confirmation can be problematic [13] since genomic fragments containing a new RE insertion could be absent in another aliquot of the same DNA sample. In many cases, the initial sample is limited that restricts the number of locus-specific PCR reactions necessary to prove the identified insertions. Here we present a new method called SeqURE (Sequencing of Unknown Retroposition Events) for the detection of young RE insertions in the human genome that overcome these limitations. The method is designed to amplify both 5′ and 3′ flanking sequences from the same DNA sample and to identify TSD in the sequenced library. We demonstrate the power of our approach on the retroelements of Alu subfamilies. We also developed the first original protocol for measuring the RE identification methods’ sensitivity. Using this protocol for Alu insertions we evaluated the sensitivity of our method of detecting Alu insertions at the level of 1 RE insertion per 1000 cells. This protocol could be easily implemented for the testing of other RE identification methods.

## Results

### Principle of the method

**Fig. 1:**
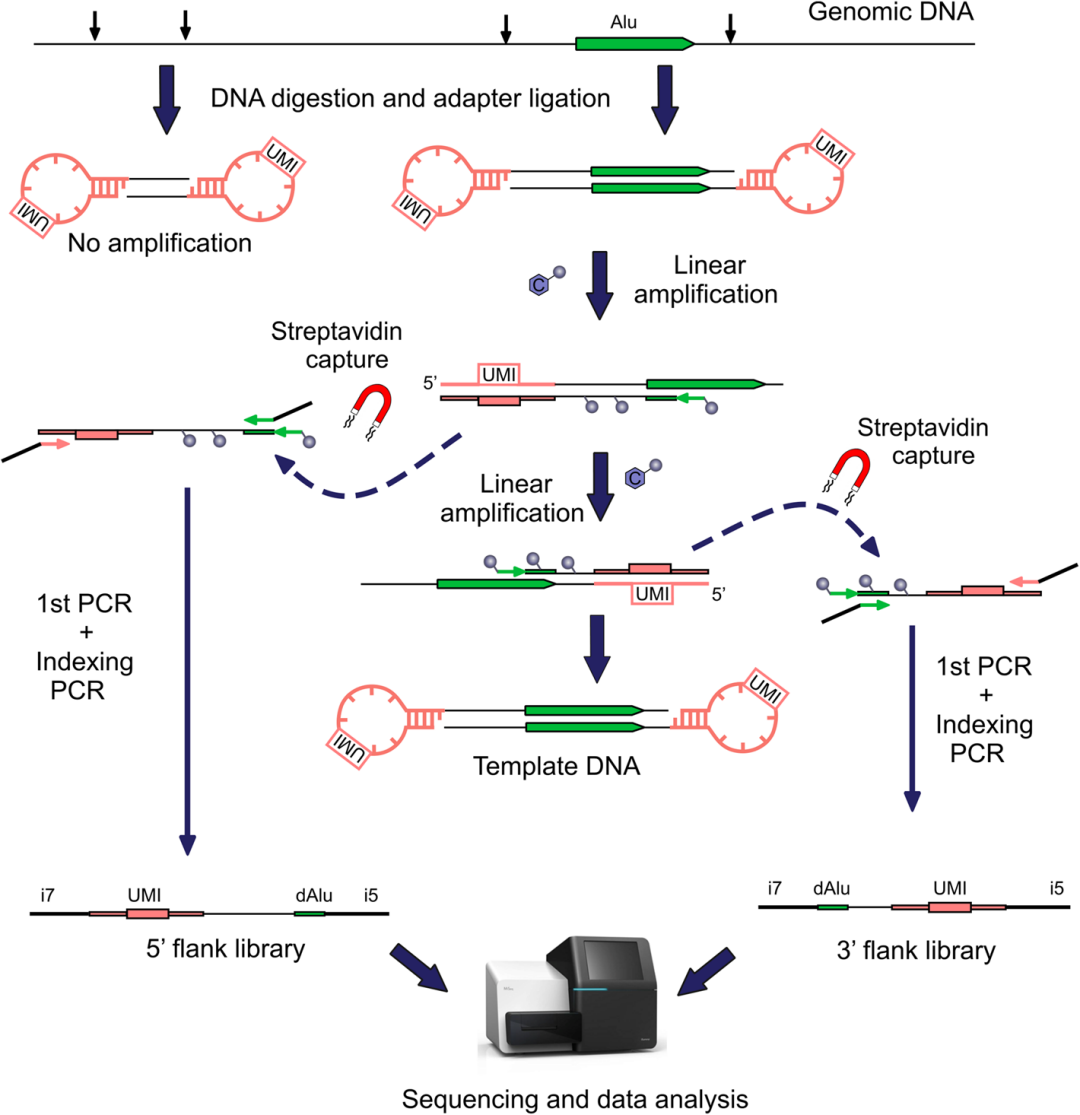
Principle of the method. Genomic DNA is digested by a mixture of restrictases that do not cut inside Alu element. After adapter ligation and linear amplification with AluYa5 or AluYb8 specific primer the copy of Alu 5′ flank is captured by streptavidin coated magnetic beads. This procedure is repeated for the 3′ flank of Alu. Captured linear amplification products are used in two stage exponential PCR (1st PCR + indexing PCR) to generate libraries for sequencing on Illumina. Remaining template DNA is used for validation in locus-specific PCR. UMI – Unique Molecular Identifier, dAlu – part of Alu repeat, i7 and i5 standard Illumina Nextera sample barcodes. Blue circles indicate biotin attached to the Alu specific primers and dCTP

**Fig. 2:**
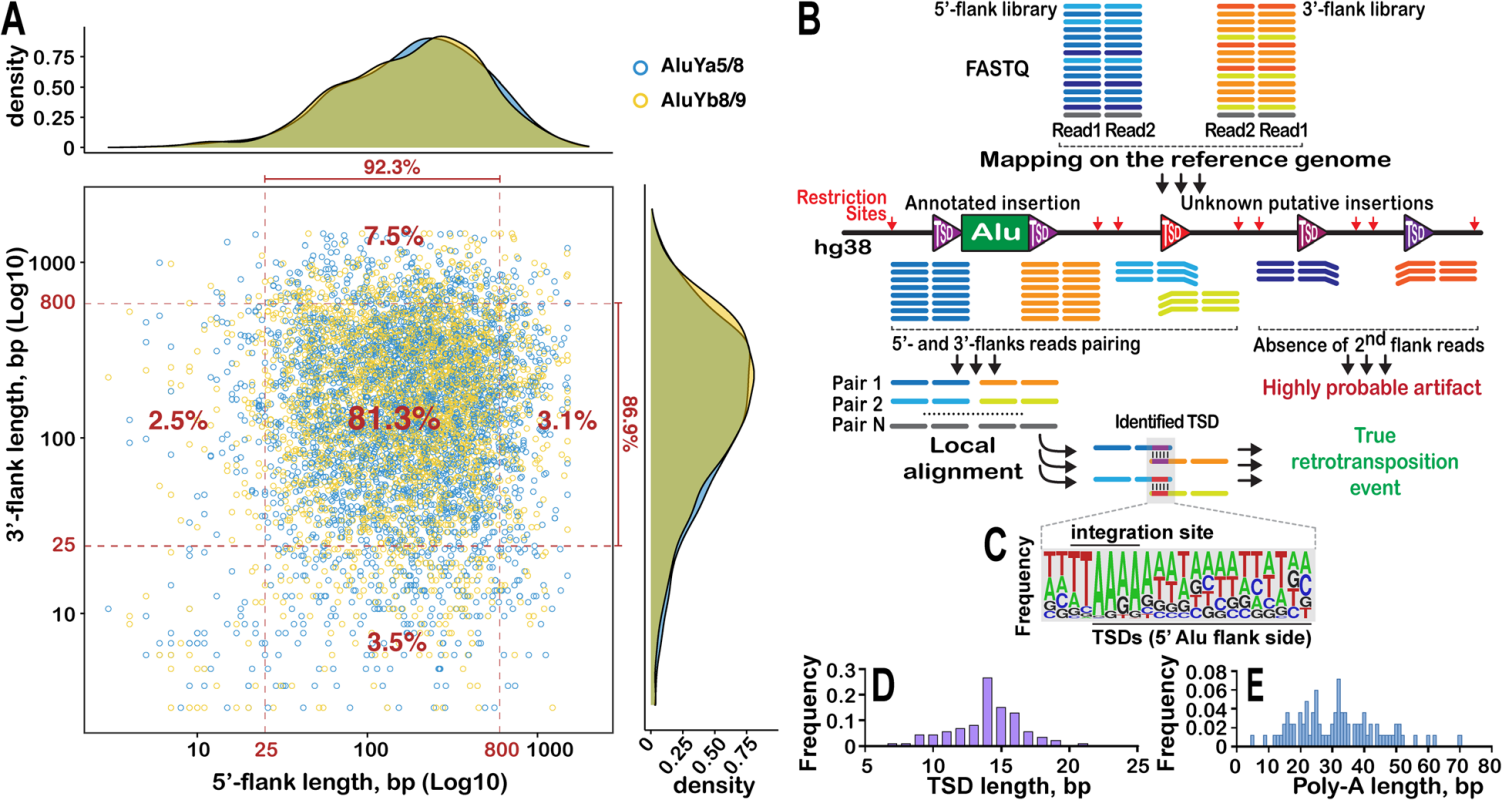
**a**. Distribution of 3′ and 5′ flank length (fragmentation by FspBI and Csp6I restrictases) of reference hg38 full-sized insertions belonging to active Alu subgroups (AluYa5 and AluYb8). 25–800 bp long flank is suitable for Illumina sequencing and correct mapping to the human genome. **b**. Scheme of TSD (Target Site Duplication) identification principle. 5′ and 3′ flank library reads are independently mapped to the reference human genome. Integration point of each insertion is situated between nearest restriction sites. Pairs of 5′ and 3′ library reads belonging to the same insertion are detected using genomic coordinates of mapped reads ends and expected nearest restriction site. **c**. Weblogo diagram of nucleotide frequencies in identified TSD and integration sites (see Additional file 2) of previously unknown Alu insertions used for the method testing. TSD identification was performed using sequences of 5′ and 3′ flank libraries. **d**. TSD length (see Additional file 2) distribution for Alu insertions used for the method testing. **e**. Poly-A tail length (see Additional file 2) distribution for Alu insertions used for the method testing

The method is based on consecutive selective amplification of both 5′ and 3′ flanking regions adjacent to the copies of retroelements belonging to young RE subfamily of interest (Fig. 1). For detection of Alu (AluYa5 + AluYa8, hereafter AluYa5 or AluYb8 + AluYb9, hereafter AluYb8) genomic DNA is fragmented by a mixture of endonucleases FspBI and Csp6I which do not cut inside the Alu element. This combination of enzymes cleaves 92% of the known AluYa5 and AluYb8 5′-flanks into fragments 25–800 bp long (Fig. 2a). On the one hand, such length is suitable for the efficient cluster generation and sequencing on Illumina, and on the other hand, in most cases it is enough for RE insertions mapping to the genome. Additionally, 87% of known AluYa5 and AluYb8 have 3′ flanks of the same length and 81% have both flanks in the range of detection. Moreover, 98% of AluYa5 and AluYb8 insertions can be identified using either one or the other flank. Thus, despite using non-random fragmentation the power of the developed approach is comparable with the methods based on random DNA fragmentation such as ME-Scan [17]. We prefer DNA digestion by site specific restrictases to random sheering. Despite this approach leads to the loss of insertions having very short or very long flank (i.e. genomic distance between the insertion point and the nearest restriction site) it has some advantages over random fragmentation for identification of rare events. First, using a restrictase with the known restriction site prevents fragmentation of the element itself which is critical for both flanks amplification (see below). Second, using of restriction enzymes allows for the identification of chimeric sequences resulting from genomic fragments ligation that can mimic real insertion events.

At the next step fragmented DNA is ligated to stem-loop DNA adapters containing Unique Molecular Identifiers (UMI) [25]. These random 10 nt oligonucleotides are used to label each molecule containing an insertion with a unique “molecular barcode”, to remove PCR duplicates and to evaluate the number of cells bearing each insertion. The stem-loop adapter structure was designed to increase the ligation efficiency and thus maximize the chance to ligate the adapter to both ends of each DNA molecule. This oligo contains sequence identical to the primer used in the first PCR reaction that prevents amplification of molecules without target Alu sequence similar to “vectorett PCR” approach. At the next stage remaining adapters are inactivated by the ligation to anti-SL oligo. This procedure prevents residual adapters from priming amplification reaction at the next step and thus artificial increasing of UMI counts.

Next, the biotinylated primer specific to AluYa5 (or AluYb8) fragment containing 3 diagnostic mutations for Ya5 (or specific 7 nt fragment for Yb8) is used for linear amplification of retroelement 5′ flanks. The biotinylated primer along with the biotinylated dCTP is used to introduce biotin into generated ssDNA molecules. After linear amplification the obtained product is denatured, the biotinylated 5′ flank copies are captured and removed from the reaction with the use of streptavidin-coated magnetic beads. Importantly, the restricted and adaptor-ligated genomic DNA template remains intact. This DNA is used for the second linear amplification with the primer complementary to the same region characteristic for AluYa5 (or AluYb8) but in the opposite direction (towards the 3′ flank of the insertion). This product is also captured by streptavidin-coated magnetic beads. The remaining initial template DNA is used to confirm insertions by locus-specific PCR. Captured linear DNA is amplified in the subsequent PCR to generate libraries of AluYa5 (AluYb8) flanks (separately for 5′ and 3′ flank). The second PCR is used for sample barcoding and generates libraries ready for sequencing on Illumina. The resulting fragments consist of a short part of the Alu repeat, adjacent genomic flank (5′ or 3′), a part of the adapter with UMI, and standard parts necessary for Illumina sequencing including sample barcodes. 3′ flanking library also includes the polyA tail of the Alu.

After sequencing, we use a custom computational pipeline [24] to map all sequenced insertions flanks to the human genome. Coordinates of insertions in compared samples (e.g. normal vs tumor tissue, mother-father-children, etc.) are matched to identify sample-specific insertions. Importantly, our pipeline is able to filter out most artificial chimeric sequences that mimic real RE insertions. At the last stage, sample-specific insertions from both 5′ and 3′ flanks libraries are matched. Matched sequences adjacent to the Alu insertion are used to identify TSD. The number of UMI for each insertion is also counted to evaluate the percentage of cells in a sample bearing each insertion.

### Development of the protocol for testing method sensitivity

To evaluate method sensitivity, we used polymorphic Alu insertions. Using our method we first identified coordinates of AluYa5 insertions in the genomes of four healthy individuals (D1, D2, D3 and D4). Then we compared these coordinates and identified in total 137 AluYa5 insertions that are absent in the genome of individual D1 and present in the genome of one of the individuals D2, D3, or D4 (see Table 1 for numbers and Additional file 1 for the detailed description of each detected insertion). Twenty five out of these 137 insertions are reference insertions present in the hg38 human genome. Eighty one AluYa5 are previously known non-reference insertions found in dbRIP (http://dbrip.brocku.ca/) or 1000 genomes project databases [26]. The remaining 31 insertions are unknown non-reference polymorphic or germline insertions discovered in this study for the first time. Next, we made a mixture of T cells containing 50,000 cells of individual D1, 500 cells of individual D2 (1%), 150 cells of individual D3 (0.3%), and 50 cells of individual D4 (0.1%) using FACS sorting. Cells of each individual were stained by antibodies with different fluorescent dyes (see Methods). Mixing was done in five replicates. Three of them where re-run on FACS to evaluate the accuracy of sorting (see Table 1, cell counts and Methods). DNA from the other two replicates was extracted and 1/10 aliquots (the equivalent of 5000 mixed cells) were used to prepare libraries of Alu Ya5 5′ and 3′ flanks with SeqURE method.

**Table 1:** Number of Alu insertions present in genomes of individuals D2-D4 and absent in the genome of individual D1

	D2 (1%)	D 3 (0.3%)	D 4 (0.1%)
AluYa5	44	45	48
Cell counts	1.11–1.42%	0.31–0.33%	0.05–0.14%

After sequencing (see Table 2 for sequencing read numbers) and data processing (as described previously in [24]) we searched for 5′ flanks of Alu insertions characteristic for genomes of individuals D2-D4 (from Table 1) in the corresponding datasets. As a result, we were able to identify 44 out of 44 insertions present in 1% (individual D2) of cells, 42–43 out of 45 insertions present in 0.3% (individual D3) of cells and 34–39 of 50 insertions present in 0.1% (individual D4) of cells (See Table 2). The insertion was considered “found” if it has at least 2 sequencing reads corresponding to its flank in the dataset. The 5′ flanking fragment length (genomic distance between insertion coordinate and the closest restriction site) in all 137 tested Alu insertions was between 30 and 529 bp.

**Table 2:** Number of individual-specific insertions found in 5′ and 3′ Alu flank libraries

	Individual 2	Individual 3	Individual 4
Found in 5′ flank library			
Replicate 1 (15,372,014 reads)	44 (44)^a^	43 (45)	39 (50)
Replicate 2 (20,116,093 reads)	44 (44)	42 (45)	34 (50)
Both Replicates	44 (44)	41 (45)	29 (50)
Found in both 5′ and 3′ flank libraries			
Replicate 1 (18,644,933 reads)	34 (36)	26 (37)	18 (36)
Replicate 2 (19,315,147 reads)	32 (36)	29 (37)	16 (36)
Both Replicates	32 (36)	23 (37)	11 (36)

^a^ - Number of expected insertions are given in parentheses

Next, we searched for the same insertions in the 3′ Alu flanks library. Obviously, a portion of these insertions have their restriction sites located too far from the integration point and their flanks are lost during amplification and Illumina sequencing. Thus, we first analyzed the distribution of restriction sites in the 3′ flanks of Alu insertions identified in the 5′ flank library. We found that 36 out of 44 Alu for individual D2 (1%), 37 out of 45 Alu for individual D3 (0.3%) and 36 out of 48 Alu for individual D4 (0.1%) have nearest 3′ restriction site in the range of 30–529 bp (same as for the 5’flank). Next, we searched the 3’flank library for the insertions that are present in both 5′ and 3′ flank libraries. As a result, we were able to find 32–34 out of 36 insertions present in 1% (D2) of cells, 26–29 out of 37 insertions present in 0.3% (D3) of cells and 16–18 out of 36 insertions present in 0.1% (D4) cells (See Table 2). Next, we compared sequences which are directly adjacent to each insertion from 5′ and 3′ side and were able to identify TSD for 87% of AluYa5 found in both 5′ and 3′ libraries (Fig. 2b, Additional file 2). For the remaining insertions the quality of the sequencing read following polyA track was insufficient to reliably identify TSD.

**Fig. 3:**
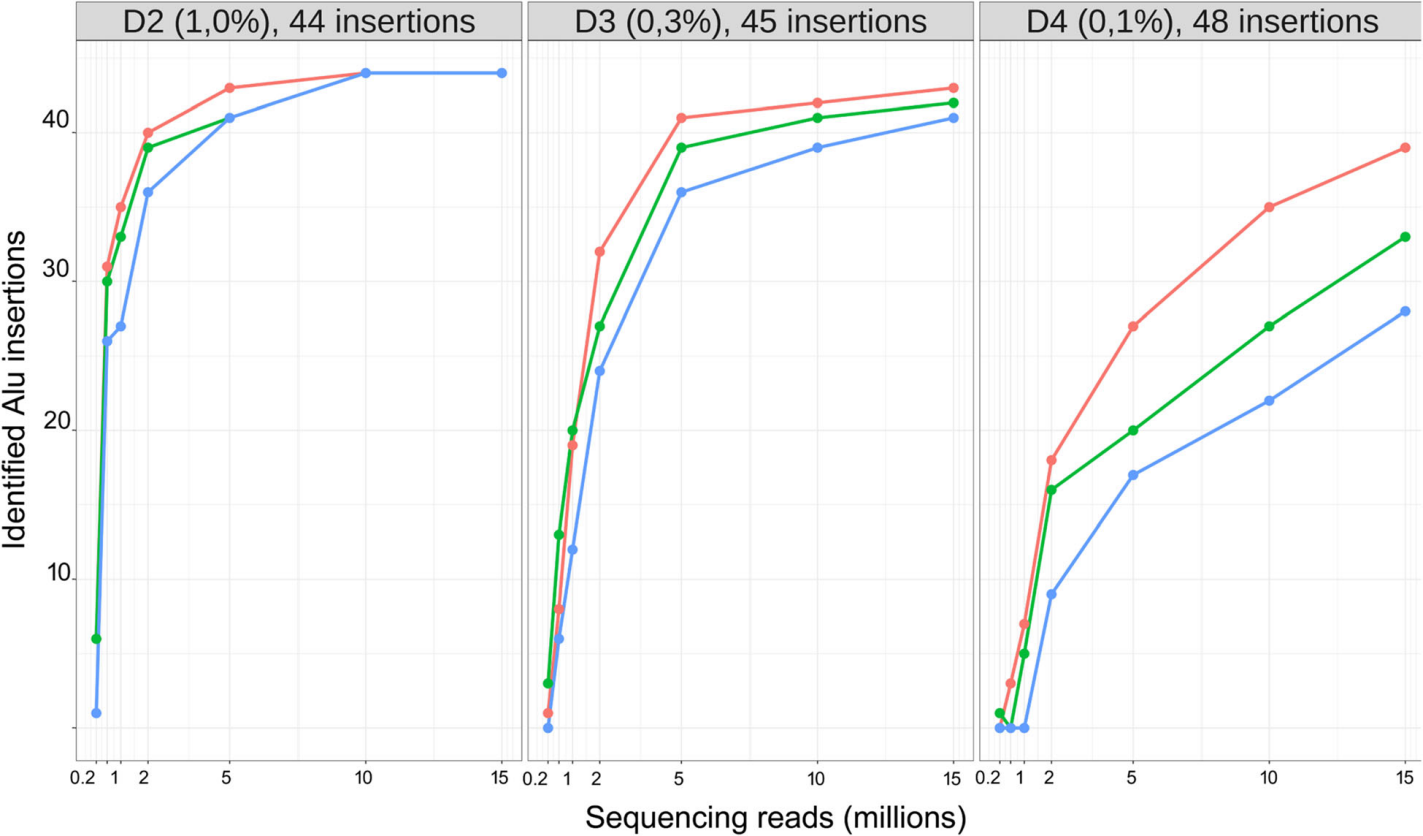
Results of downsampling experiments on the 5′ Alu flank datasets. Number of raw sequencing reads used in each sampling experiment is indicated on X axis. Red line – the number of individual specific Alu insertions found in replicate 1, green line – in replicate 2, and blue line in both replicates simultaneously

To evaluate the sequencing depth that is required for confident identification of Alu insertions we performed downsampling experiments on the 5′ flanks Alu datasets. For this purpose, we randomly selected 15, 10, 5, 2, 0.5, 0.2, and 0.1 millions of raw sequencing reads from 5′ Alu flank datasets obtained from two mixed cells replicates and searched for the individual-specific insertions (see Fig. 3). The insertion was considered “found” if it had at least 2 sequencing reads corresponding to its flank in the dataset.

For 1% of cells, we found 100% insertions in both replicates with a sequencing depth of 10 million reads. The vast majority (39–40 out of 44 in a single replicate and 36 in both replicates) of insertions present in 1% of the cells in a sample can still be found with sequencing depth of 2 million reads. As expected, for the cells with 10 times lower concentration (0.1%) even 15 million of reads is not enough to catch 100% of the insertions, however, 28 out of 48 (approximately 60%) are repeatedly found in both replicates. Around 20% of insertions are still repeatedly detectable with sequencing depth of 2 million in both replicates (Fig. 3).

**Fig. 4:**
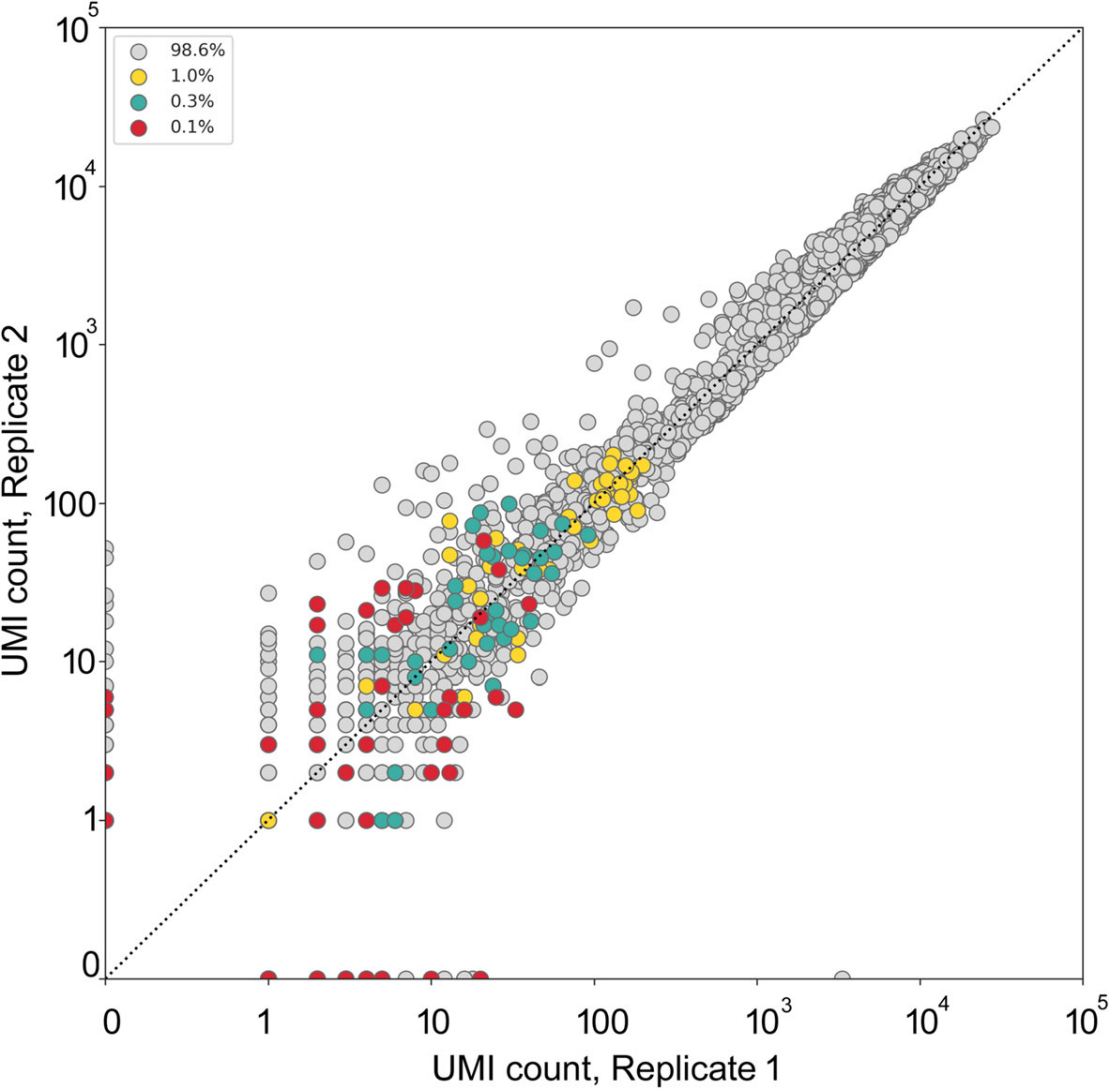
Reproducibility between replicates. Grey dots – known fixed and polymorphic insertions of individual D1, yellow dots – individual specific insertions of D2 (1%), green dots – individual specific insertions of D3 (0.3%), red dots – individual specific insertions of D4 (0.1%)

Reproducibility of the method was evaluated by the comparison of two independent replicates (independent cell mixtures). We plotted normalized UMI count in replicate 1 versus UMI count in replicate 2 for each of the identified Alu insertion from individuals D1-D4 (Fig. 4). Overall correlation coefficient between two replicates was *r*^*2*^ = 0.97. For the insertions present in minor percentage of cells it was *r*^*2*^ = 0.75, *r*^*2*^ = 0.43, and *r*^*2*^ = 0.17 for 1, 0.3 and 0.1%, respectively.

Initial DNA template (restricted genomic DNA with ligated adaptors) that left after two rounds (5′ and 3′ flank) of linear amplification and capture was used as a template for locus-specific PCR. We selected 10 non-reference Alu insertions which have a distance between 5′ and 3′ flanks restriction sites in the range 400–600 bp. Next, we removed short (100–300 bp) template DNA molecules from the mixture by magnetic beads. This procedure depletes DNA fragments that do not contain Alu insertions (empty allele) originating from the genome of individual D1. After that, we performed locus-specific PCR with the primers corresponding to unique flanking regions of each insertion. We observed PCR product of expected length for 4 out of 4 insertions present in the genome of D2 (1% of cells), for 3 of 4 insertions present in the genome of D3 (0.3% of cells) and for 1 of 2 insertions present in the genome of D4 (0.1% of cells). Obtained PCR products were sequenced by Sanger method which in all cases allowed to confirm the insertions by the alternative method and were able to identify TSD indicating true Alu insertions.

### Identification of tumor-specific Alu insertions in colorectal cancer samples

Using our method, we performed a search for cancer-specific Alu Ya5 and AluYb8 insertions in 6 paired (tumor/normal) colorectal cancer samples. Libraries of 5′ and 3′ flanks were prepared using SeqURE method and sequenced. For each 5′ flank library we obtained a minimum of 2,173,258 reads and the difference between tumor and normal samples in each pair was less than 2-fold. After data processing and artifact filtration, we compared lists of Alu insertion coordinates in paired normal and tumor samples. Only the insertions with 2 or more different UMI present in the tumor sample and absent in corresponding paired normal sample were considered tumor-specific candidates. We identified 10 potential tumor-specific Alu insertions in the 5′ flank libraries of 5 out of 6 tumor samples (see Table 3). Using the same approach as for the method sensitivity assay, we searched for corresponding sequences in 3′ flank libraries. For 7 out of 10 candidates identified in the 5′ flank libraries, we found no corresponding sequences in the 3′ flank library. These candidates are most probably artifacts that are not filtered by our pipeline. For the other 3 candidates, we found corresponding 3′ sequences in both tumor and normal libraries. These insertions could represent non-reference polymorphic or germline insertions present in both normal and tumor cells but detected only in the tumor 5′ flank library due to insufficient sequencing depth. For 8 candidate insertions, we designed primers and performed locus-specific PCR with the initial template as described above. In all cases, we were unable to detect PCR products of expected length confirming that candidate Alu insertions from 5′ flank libraries were artifacts.

**Table 3:** Candidate tumor-specific Alu insertions

Sample	RE family	Chromosome	Position	UMI count in 5′ flank library	3′ flank Length	UMI count in 3′ flank library	PCR validation
CCC8	AluYa5	8	137,710,278	4	44	0	No
	AluYa5	1	39,888,222	2	544	0	No
	AluYb8	5	94,414,349	2	218	0	No
CCC9	AluYb8	5	77,457,780	2	89	148(+ 397)^a^	–
	AluYb8	7	43,035,359	3	137	2(+ 1)^a^	No^b^
CCC11	AluYa5	8	80,855,361	2	212	456(+ 283)^a^	–
	AluYa5	4	103,090,594	2	20	0	No
	AluYa5	1	9,512,325	2	108	0	N/A
CCC13	AluYa5	16	70,789,164	2	123	0	No
CCC14	AluYa5	10	80,758,919	2	423	0	No

^a^ also found in corresponding normal sample

^b^ - PCR product detected in both tumor and normal samples

N/A – impossible to design primers due to insertion into other repetitive sequences

To further proof the developed copy-capture approach we identified cancer-specific insertions of another family – L1HS in the same set of paired tumor/normal samples. As amplification and sequencing of 5’flanking regions of L1 insertions is yet challenging we generated only the library of 3′ flanks, using the same experimental approach as for Alu 3′ flank libraries. Genomic DNA was digested with another pair of restriction enzymes and ligated to adapted with the same pan-handle like structure (see methods for details). After linear amplification with L1HS specific biotinylated primer 3′ flank copies were extracted with streptavidin-coated magnetic beads and amplified in the subsequent 2 round PCR to generate ready-for-sequencing sample-barcoded libraries. Same as for Alu libraries, template DNA stays intact and is used for validation by locus-specific PCR. For each library we obtained a minimum of 219,970 reads and the difference between tumor and normal samples in each pair was less than 2-fold. In six paired samples combined we found 1119 L1HS insertions including 854 reference insertions (hg38), 188 previously known non-reference insertions (present in 1000 genomes, dbRIP or euL1db [27]) and 77 previously unknown non-reference insertions. We identified 10 tumor-specific candidate insertions in 5 out of 6 tumor/normal sample pairs (see Table 4). For 10 candidate insertions, we designed primers and performed locus-specific PCR with the initial template (see Methods). For 4 out of 10 insertions we obtained PCR product of expected length with tumor but not corresponding normal sample. For 3 other insertions we obtained PCR product in both tumor and normal samples and for the rest of insertions (*n* = 3) we failed to get PCR product. The obtained PCR products for 4 tumor-specific insertions were sequenced by Sanger method. In all cases we confirmed tumor-specific L1HS insertions.

**Table 4:** Candidate tumor-specific L1HS insertions

Sample	Chromosome	Position	UMI count in 3′ flank library	PCR validation
CCC8	8	137,421,701	3	No^b^
	X	144,010,198^a^	4	No^b^
CCC9	5	28,280,060	13	Yes
	8	16,154,249	3	No^b^
ССС10	20	53,163,810	2	No
	5	151,055,147	2	No
	8	128,834,433	2	No
CCC11	3	138,985,467	7	Yes
	3	155,181,290	16	Yes
CCC13	X	38,440,949	13	Yes

^a^ Possibly 3′ transduction

^b^ - PCR product detected in both tumor and normal samples

## Discussion

In the recent years it is becoming widely accepted that RE transcriptional and transpositional activity can play a significant role in cancer. New insertions can disrupt cellular genes (including those involved in malignant transformation) either by direct integration into a gene [28, 29] or via recombination-mediated chromosomal rearrangement [16]. A recent study by Cajuso et al. [30] showed that the amount of cancer-specific L1 insertions in colorectal cancer correlates with poor prognosis. With the growing capacity of modern sequencing machines, whole genome sequencing price is decreasing. However, the discovery of somatic variants including somatic insertions of RE in cancer cells still requires high sequencing coverage making analysis of hundreds of samples quite expensive. The identification of insertions present in a minor fraction (1–10%) of tumor cells is not possible at all at the commonly used whole-genome sequencing depth. These insertions could be of particular interest as they can characterize a subclone that is functionally different from other tumor cells and can be a source of relapse in course of therapy. Thus, the development of cheap, sensitive, and simple methods for tumor-specific RE identification is desired. Here we present a method that combines high sensitivity, reliability and requires a moderate number of sequences per sample thus allowing the screening of hundreds of samples at a reasonable cost.

We use a modified selective amplification approach [22, 31] to generate the library of Alu flanks. As was shown previously [21] and in the current study selective amplification-based methods are very specific and have high (90% and more) target sequence (flanks of young Alu or L1) content in the resulting library. This approach gives an approximate enrichment of 3000-fold in target RE flanking sequences compared to whole-genome shotgun libraries. The resulting library for individual D1 includes 2367 (2046 reference hg38 and 321 non-reference) or 3042 (2617 reference hg38 and 425 non-reference) previously known AluYa5 insertions depending on sequencing depth (632,732 and 35,488,107 sequencing reads respectively). This number of insertions (3042) makes 91.5% of the average number of full-size AluYa5 insertions (3324) detected in an individual genome by whole genome sequencing (evaluated from 1000 genomes [26, 32]).

The significant drawback of this approach is the inability to identify TSD – the hallmark of the retrotransposition event. As a result, even with very high sensitivity and vigorous artifact filtration pipelines, many potential insertions are not confirmed by the locus-specific PCR. This limitation can be overcome by the amplification of both flanking regions (5′ and 3′). However, this approach is not reliable when detecting insertions present in a minor fraction of cells if different aliquots of DNA from the same sample are used for library preparation and validation. Here we consecutively amplify both adjacent genomic regions of each insertion from the same template which significantly increases the chance to retrieve both flanks and identify TSD. In addition, sequence of one flank is used to validate the insertion found in the library of another flank: when we know the insertion coordinate from one library we can predict the length of another flank as we use restrictase for DNA fragmentation. If the corresponding sequence has suitable length for amplification and sequencing but not found in another flank library – this indicates false insertion. Moreover, template genomic DNA used for libraries preparation stays intact and can be used for locus-specific PCR to confirm insertions. Using spike-in controls we show that this approach can indeed produce reliable results. Most of the insertions that have corresponding sequences in 5′ and 3′ flank libraries were confirmed by locus-specific PCR from the same DNA template. We also directly characterized the sensitivity of the method. We show that the majority of insertions present in as little as in 1% of cells can be reliably (in two replicates) identified with the sequencing depth of only 2 million of 150 + 150 paired reads (of 0.6 Gb) per sample. This corresponds to 0.2x human genome coverage. It should be mentioned that uneven amplification efficiency of different flanking sequences results in different sequencing coverage for each particular RE insertion. As it was shown previously [21] amplification efficiency is proportional to flank length and also depends on other structural features of RE and its flanking region. This amplification and sequencing bias can be considered as a potential limitation of the proposed method as a portion of insertions is lost. However, such bias is characteristic for all PCR based methods. The proposed method is easily adapted to high-throughput formats and could be automated which gives a very powerful and cost-effective tool for researchers aiming to study hundreds of samples for new RE insertions. This method can be combined with the previously designed approach to advanced enrichment for rare somatic insertions [21]. Such combination could dramatically increase reliability and decrease the cost of somatic RE identification assay. The developed protocol for testing sensitivity of RE detection methods could help researchers in the field to evaluate and elevate the efficacy of existing and developing methods.

Unlike L1 insertions, tumor-specific Alu insertions are rare in cancers including colorectal cancer [33]. As a proof of concept, we searched for such events in 6 paired colorectal tumor/normal samples. With the sequencing depth sufficient for the identification of most insertions present in 1% of cells we detected 10 potentially tumor-specific AluYa5 insertions in the 5′ flank library. These candidate insertions have 2–4 UMI indicating their presence in a minor percentage of tumor cells. None of these candidates were confirmed in the corresponding 3′ flank library or were found in both tumor and normal sample 3′ libraries. Concordantly, none of these insertions were validated by locus-specific PCR. This finding indicates high reliability of the method: comparing sequences obtained for both flanks from the same template allows distinguishing true insertions from false candidates.

To further validate the method, we searched for tumor specific L1HS insertions in the same set of samples using the same methodological approach. We identified 10 candidate insertions with UMI counts ranging from 2 to 16 and fully confirmed 4 of them by orthogonal approach. These results indicate successful application of our method on real samples, and at the same time, demonstrate the necessity for sequencing of both flanks for reliable tumor-specific RE identification. Additionally, the identified tumor specific L1HS insertions indicate that at least 3 out of 6 analyzed colorectal tumor samples were subjected to retropositional activity. At the same time, we accurately showed absence of tumor specific Alu insertions in all 6 samples. Taken together, these facts indicate that Alu insertions in cancer occur much rarer than L1 and active L1 machinery alone is insufficient for Alu retropositional activity.

## Conclusion

Most of the previously described techniques for targeted RE capture like RC-seq [34] use original DNA molecules which become unavailable after library preparation. Here we developed the new capture method that extracts copies of target molecules saving original DNA for downstream application such as validation assay, SNV sequencing or DNA methylation analysis. The described principle can be easily adapted for library enrichment by other target molecules after or instead of RE capture just by adding new target primers to the reaction. Thus, the proposed approach is characterized by high level of flexibility that can cover requirements of modern experimental demands for simultaneous analysis of different items or parameters in the same biological sample.

## Materials and methods

### Sample collection and DNA isolation

Six colorectal cancer samples were obtained during R0 partial colectomy from patients with stage II or III colorectal adenocarcinoma (5 patients) or carcinoma in situ (1 patient) treated at the A.N. Ryzhikh National Medical Research Centre for Coloproctology. The study was approved by the local ethical committee and all the patients gave standard informed consent. Tumor and normal tissue fragments were taken under the supervision of a pathomorphologist in the shortest possible time after intestinal resection (not more than 30 min). DNA was extracted with QIAamp DNA Mini Kit (Qiagen) according to manufacturer’s protocol.

PBMC of four healthy donors were isolated from peripheral blood by standard Ficoll-Paque (PanEco, Russia) centrifugation protocol. T-cells from different individuals were stained by the following antibodies: individual D2 CD3-eFluor450 (UCHT1, eBioscience), individual D3 - CD3-FITC (UCHT1, eBioscience), individual D4 CD3-PC5 (UCHT1, Beckman Coulter). Cells of individual 1 were not stained. FACS sorting was performed in 5 replicates by BD FACS Aria III. Cells were gated based on side and forward scatter. Each cells mixture contained 50,000 cells of individual D1, 500 cells of individual D2, 150 cells of individual D3 and 50 cells of individual D4. After sorting three out of five aliquots were resorted to evaluate the resulting number of cells in the mixture. DNA from sorted cells was isolated using QIAGEN DNeasy Blood and Tissue Kit (Qiagen).

### Alu library preparation and sequencing

To avoid possible contamination, we first prepared libraries from cell mixtures and sequenced them. After that we made libraries from DNA of individuals D1-D4 separately to identify individual-specific Alu insertions present in the genome of individuals D2, D3 or D4 and absent in the genome of individual D1.

Thirty ng of genomic DNA was digested by incubation in 10 μl of 1x FD buffer with 5 U of FspBI and 5 U of Csp6I (all Thermo Fisher Scientific) for 30 min at 37 °C. For adapters ligation fragmented DNA was diluted by 20 μl of 1x FD Buffer with 20 μmol of ATP (Thermo Fisher Scientific), 50 pmol of SL-adapter (see Additional file 3 for oligo sequences), 10 U of T4 DNA ligase (Thermo Fisher Scientific) and incubated at 22 °C for 30 min. Next, 50 pmol of antiSL-adapter, additional 5 U of FspBI and Csp6I endonucleases were added and the mixture was incubated at 22 °C for 30 min and 37 °C for 30 min. AntiSL-adapter inactivates SL-adapters and endonucleases decrease the number of ligation chimeric molecules. The reaction mixture was purified with 0.8 V of AmPure XP beads (Beckman Coulter) and eluted directly in 15 μl of linear amplification reaction mixture containing 1x Encyclo Buffer, 1x Encyclo polymerase, 200 μM of each dNTP (all Evrogen, Russia), 20 μM of biotin-16-dCTP (Jena Bioscience, Germany), 0.2 μM of 5′- flank oriented AluYa5 specific biotinylated primer (Additional file 3). The linear amplification profile was: 94 °C for 3 min, followed by 30 cycles of 20 s at 94 °C, 20 s at 65 °C and 1 min at 72 °C with ramp rate 1 °C/s. After amplification the product was mixed with 3 μl of MyOne Streptavidin C1 Dynabeads (Thermo Fisher Scientific) resuspended in 1x Encyclo buffer and incubated for 15 min at room temperature with permanent rotation. Linear amplification product was eluted by 10 μl of mQ water from streptavidin coated beads, purified by 1.5 V of AmPure XP beads and used for subsequent exponential PCRs. The supernatant was supplemented by 1x Encyclo polymerase, 20 μM of biotin-16-dCTP and 0.2 μM of AluYa5 specific 3′- flank oriented biotinylated primer and used for the 2nd linear amplification to obtain linear DNA fragments of opposite (3′-) Alu flanks. The amplification profile, capture and purification condition were the same as for 5′- flank libraries. The remaining supernatant containing restricted and ligated template genomic DNA was used for the downstream validation of detected insertions by locus-specific PCR (see below). The captured linear amplicons (copies) were used in separate (for each flank) 25 μl PCR reactions containing 200 μM of each of dNTP, 0.2 μM of the Alu specific primer (see Additional file 3), adapter specific primer korNxtSt19ok and 1x Encyclo polymerase in 1x Encyclo Buffer. The amplification profile was: 2 min at 94 °C followed by 10 cycles of 20 s at 94 °C, 20 s at 60 °C, 1 min at 72 °C with ramp rate 1 °C/s. One μl of the obtained PCR product was used in the second 25 μl PCR reaction containing 200 μM of each dNTP, 0.2 μM of each Nextera Indexing primers and 1x Encyclo polymerase in 1x Encyclo Buffer amplified for 12 cycles using the same amplification profile as for the first PCR. Second PCR products were purified with AmPure XP beads, mixed equimolarly and sequenced on Illumina NextSeq paired end 150 + 150. Reference libraries from single individuals were sequenced separately on MiSeq paired end 150 + 150.

DNA from colorectal cancer samples was processed exactly in the same way with two additional libraries containing AluYb8 flanks (see Additional file 3) prepared for each sample pair. Libraries were sequenced on Illumina NextSeq paired end 150 + 150.

### L1HS library preparation and sequencing

Thirty ng of genomic DNA was digested by incubation in 10 μl of 1x FD buffer with 5 U of FspBI and 5 U of TaqI (all Thermo Fisher Scientific) for 30 min at 37 °C and 30 min at 65 °C. For adapters ligation fragmented DNA was diluted by 20 μl of 1x FD Buffer with 20 μmol of ATP (Thermo Fisher Scientific), 50 pmol of SL-adapter-2 (see Additional file 3 for oligo sequences), 10 U of T4 DNA ligase (Thermo Fisher Scientific) and incubated at 22 °C for 30 min. Next, 50 pmol of antiSL-adapter-2, additional 5 U of FspBI and TaqI endonucleases were added and the mixture was incubated at 22 °C for 30 min and 37 °C for 30 min. The reaction mixture was purified with 0.8 V of AmPure XP beads (Beckman Coulter) and eluted directly in 15 μl of linear amplification reaction mixture containing 1x Encyclo Buffer, 1x Encyclo polymerase, 200 μM of each dNTP (all Evrogen, Russia), 20 μM of biotin-16-dCTP (Jena Bioscience, Germany), 0.2 μM of 3′- flank oriented L1HS specific biotinylated primer (Additional file 3). The linear amplification profile was: 94 °C for 3 min, followed by 30 cycles of 20 s at 94 °C, 20 s at 55 °C and 1 min at 72 °C with ramp rate 1 °C/s. After amplification the product was mixed with 3 μl of MyOne Streptavidin C1 Dynabeads (Thermo Fisher Scientific) resuspended in 1x Encyclo buffer and incubated for 15 min at room temperature with permanent rotation. Linear amplification product was eluted by 10 μl of mQ water from streptavidin coated beads, purified by 1.5 V of AmPure XP beads and used for subsequent exponential PCRs as described for Alu using L1-specific primer (see Additional file 3) and 18 cycles PCR for indexing.

The supernatant was supplemented by 1x Encyclo polymerase, 20 μM of biotin-16-dCTP and 0.2 μM each of L1 multiplex primer set with locus specific primer (designed after 3′ flank sequencing) and used for the 2nd amplification to obtain DNA fragments of opposite (5′) L1HS flanks. The amplification profile, capture and purification condition were the same as for 3′- flank libraries. The captured amplicons (copies) were indexed at the same condition as 3′ flanks amplicons, purified with AmPure XP beads, mixed equimolarly and sequenced on Illumina MiSeq paired end 150 + 150.

### Identification of individual-specific insertions

Raw sequencing reads were processed as described previously [24]. As a result, we obtained a metatable containing a list of identified AluYa5 insertions in all four individuals with their genomic positions and read counts for each of the four individuals. Based on read counts for known fixed AluYa5 insertions we identified the value of first quartile (Q1) in each individual dataset. Then, using the metatable, we found polymorphic insertions that had a read count > = Q1 in one of the individuals D2, D3 or D4 dataset and 0 reads in all other individuals. To validate the insertions found by the computational pipeline, we searched for their flanking sequences in raw sequencing reads allowing for 1 mismatch.

### Validation of RE insertions by locus-specific PCR

Primers for the specific genomic regions were designed with primer-blast and GeneRunner programs. Twenty-five μl PCR reactions containing 200 μM of each of dNTP, 0.2 μM of each of forward and reverse locus-specific primers (Additional file 3) and 1x Encyclo polymerase in 1x Encyclo Buffer. The amplification profile was as follows: 2 min at 94 °C followed by 35 cycles of 20 s at 94 °C, 20 s at 60 °C, 1 min at 72 °C.

## Supplementary Information

**Additional file 1: Supplementary Table 1.** A detailed description of individual-specific Alu insertions.

**Additional file 2: Supplementary Table 2.** TSD identification.

**Additional file 3: Supplementary Table 3.** Oligonucleotide sequences used for library preparation and locus-specific PCR.

## Data Availability

The sequence reads are available from the Sequence Read Archive (SRA) under the accession number PRJNA657558.
